# Organoid-based chemical approach to dissect the mechanism controlling cellular dynamics

**DOI:** 10.1093/jmcb/mjz100

**Published:** 2019-10-22

**Authors:** Lauretta A Lacko, Shuibing Chen

**Affiliations:** Department of Surgery, Weill Cornell Medical College, New York, NY 10065, US

**Figure 1:**
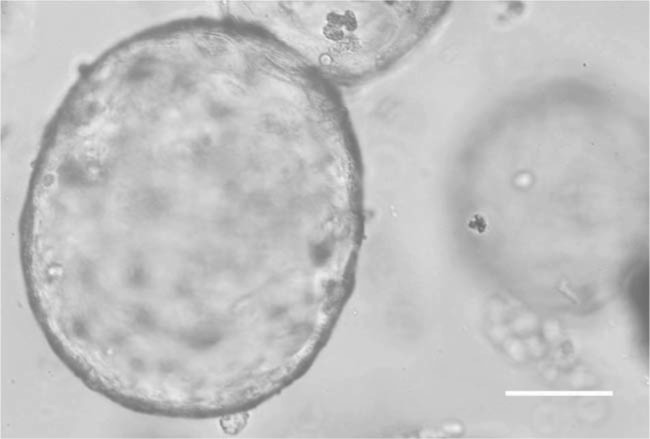
Phase contrast image of typical 3D mouse gastric organoids from 5-day culture, which exhibits functional robustness to the physiological stimuli, mitotic modulator, and reversibility of excitation–relaxation coupling. The establishment of functional 3D organoids provides a unique platform for modeling parietal cell physiology, stem cell division, and microbe–host interaction. Scale bar, 50 μm. Image provided by Liu et al. (2020).

Organoids are self-organizing *in vitro* three-dimensional (3D) tissue cultures containing multiple types of cells. Compared to traditional two-dimensional (2D) cell culture systems, 3D organoids better replicate the architecture, complexity, and physiology of an organ (Figure 1). In addition, 3D organoids are easier to scale up and more cost-efficient when compared to animal models. In the past decade, organoid technology has risen rapidly and become increasingly more prevalent among researchers. They can be cultured long-term and are easily amenable to genetic, molecular, and cellular analyses. Organoids can be derived from embryonic stem cells, induced pluripotent stem cells, adult stem cells, and tumor cells or primary tissues. Currently, 3D organoids have been created for multiple organs, including the brain, lung, stomach, small intestine, colon, pancreas, kidney, liver, prostate, etc.

Organoids are now firmly established as a critical tool to investigate normal and pathological processes in both basic science and translational clinical research. Firstly, organoids have been widely applied to explore human organ development and investigate the role of particular genes and cells. The precise role of specific genes and/or genetic mutations in organogenesis, lineage specification, and cell fate decisions have been determined using organoids derived from genetically engineered mice (Boj et al., 2015) or genetically modified human organoids (Artegiani et al., 2019). Moreover, cellular behaviors and interactions during development and disease progression can be visualized in 3D organoids using gene-editing approaches to establish reporters. Furthermore, the *in vitro* culture of 3D organoids makes it easy to manipulate the stem cell niche components to understand the role of signaling pathways in stem cell maintenance or differentiation.

Organoids also provide a robust platform to model disease progression. Significant progress has been made using 3D organoids to model infectious diseases such as Zika virus (brain organoids; Qian et al., 2017) and influenza (lung organoids; Ciancanelli et al., 2015). Using 3D organoids also provides a relevant model that surpasses several ethical issues, as demonstrated by the studies examining Zika virus infection, where the differences in mouse and human biology and the lack of fetal neural tissues provided barriers to understanding its pathology. In addition, cancer research has seen significant progress using organoids. Studying 3D organoids derived from patients allows investigation into the heterogeneity of different cancers and the molecular mechanisms that control disease progression (Sachs et al., 2018).

Finally, organoids provide a disease-relevant and cost-efficient platform to evaluate drug efficacy, toxicity, or even drug screening in a context-dependent manner. The context includes patient’s genetics, metabolism, and even microbiota (Yao and Smolka, 2019). Many efforts have been put to biobank cancer organoids, which can be used to accurately predict drug responses, efficacy, and toxicity, and guide optimal treatment strategies for precision medicine (Kondo and Inoue, 2019; Takahashi et al., 2019). The identified small molecules can also be used as a chemical tool to dissect the molecular mechanism controlling biological processes.

In this issue, Liu et al. (2020) reported a human 3D gastric organoid-based platform to model the physiology of cell division control. This process proves difficult to study in 2D as epithelial cell chromosome segregation is not accurately recapitulated without the 3D structure. Using light sheet microscopy imaging analyses, several Food and Drug Administration (FDA)-approved drugs and chemical inhibitors of mitosis were examined. Two CENP-E kinesin chemical inhibitors with distinct structures and mechanisms of action, including syntelin (Ding et al., 2010) and GSK923295 (Wood et al., 2010), were surprisingly identified to perturb cell metaphase–anaphase transition. The function o CENP-E motor in chromosome congression in metaphase alignment was extensively characterized (Wood et al., 1997; Yao et al., 2000; Mao et al., 2003); however, its involvement beyond metaphase came as a surprise. To probe if the post-metaphase involvement of CENP-E is valid, Liu et al. (2020) synchronized mitotic cells at metaphase to satisfy the requirement of CENP-E in metaphase alignment followed by a chemical inhibition of CENP-E at metaphase–anaphase transition. Further high-resolution real-time imaging analysis confirmed that CENP-E-inhibited cells undergo central spindle splitting and chromosome instability phenotype. Using biotinylated syntelin as an affinity matrix, PRC1 was identified to form a complex with CENP-E in mitotic cells. Syntelin, which blocks CENP-E in metaphase, prevents accurate central spindle assembly by perturbing temporal assembly of PRC1 to the midzone. Together, this study reveals a novel role of CENP-E in temporal control of central spindle assembly and demonstrates 3D gastric organoids as a unique model to study cellular dynamics *in vitro*. Their findings demonstrate a new function of CENP-E in mitosis. This work establishes a foundation for temporal control of CENP-E function using chemical probes such as syntelin in cell division, which is broadly applicable to experimental probing of CENP-E in other dynamic cellular processes in 3D organoids.

In summary, Liu et al. (2020) demonstrate that 3D gastric organoids are a unique model system to discover the molecular mechanisms underlying context-dependent cell division control in the 3D tissue environment. The focused chemical screen facilitated the uncovering of a novel role of PRC1 protein to bind to and regulate CENP-E in the metaphase–anaphase transition. The current study validates the power of 3D organoids and chemical screening as a model to explore the detailed mechanism controlling cell division.
